# Recyclable Palladium-Polysiloxane Catalyst with Ultra-Low Metal Leaching for Drug Synthesis

**DOI:** 10.3390/polym17223066

**Published:** 2025-11-19

**Authors:** Ekaterina A. Golovenko, Polina P. Petrova, Dmitrii V. Pankin, Sergey V. Baykov, Vadim Yu. Kukushkin, Vadim P. Boyarskiy, Regina M. Islamova

**Affiliations:** Institute of Chemistry, St. Petersburg State University, 7/9 Universitetskaya Nab., St. Petersburg 199034, Russia; ekaterina.golovenko@spbu.ru (E.A.G.); st107479@student.spbu.ru (P.P.P.); dmitrii.pankin@spbu.ru (D.V.P.); sergei.v.baikov@yandex.ru (S.V.B.); v.kukushkin@spbu.ru (V.Y.K.)

**Keywords:** palladium-containing polysiloxanes, carbon support, reusable catalysts, Sonogashira reaction, Heck reaction, Suzuki reaction, DFT calculations

## Abstract

A carbon-supported palladium-containing polysiloxane macrocatalyst (Pd-PDMS) was developed for pharmaceutical-grade cross-coupling reactions. The catalyst demonstrates exceptional year-long stability at room temperature while maintaining full catalytic activity. Pd-PDMS efficiently promotes three pharmaceutically relevant reactions: Suzuki coupling (80% yield), copper-free Sonogashira coupling (90% yield at 55 °C), and Heck coupling (80% yield at 90 °C). The copper-free Sonogashira protocol eliminates toxic copper cocatalysts, phosphine ligands, and organic bases while operating under mild conditions. Most significantly, palladium contamination in products reaches ultra-low levels of 22 ppb (Sonogashira, Suzuki) and 167 ppb (Heck), representing a 60–450-fold improvement over European Medicines Agency requirements (10 ppm). The catalyst exhibits excellent recyclability without activity loss over multiple cycles, with simple washing protocols between uses. Scanning electron microscopy and X-ray photoelectron spectroscopy confirmed uniform Pd-PDMS coating on carbon fibers, while density functional theory calculations revealed specific coordination interactions between the palladium complex and carbon support at 3.26 Å distance. This convergence of pharmaceutical-grade metal contamination control, exceptional stability, and multi-reaction versatility establishes a significant advancement for sustainable cross-coupling catalysis in pharmaceutical applications.

## 1. Introduction

Heterogeneous palladium-containing catalysts are extensively used on an industrial scale in catalytic reactions such as chemoselective hydrogenation [1,2], reduction in various functional groups using hydrogen donors [3,4], debenzylation and deallylation reactions [5], C–C cross-couplings [6]. The latter play an important role in the synthesis of compounds exhibiting various types of biological activity and medicines [7], providing access to a robust class of reactions such as Suzuki, Sonogashira, and Heck couplings [8,9,10].

The Suzuki reaction is actively used in the pharmaceutical industry [8] for the synthesis of different medicines such as angiotensin receptor blockers, non-steroidal anti-inflammatory drugs, kinase inhibitors, and protease inhibitors [11]. No less significant for medical applications are Sonogashira and Heck reactions, which are extensively utilized for the synthesis of antioxidant compounds, antidiabetic, antibacterial, and anticancer medicines [10,12]. The copper-free Sonogashira reaction has emerged as a particularly important methodology, with recent mechanistic studies revealing a palladium-palladium transmetalation pathway that operates without the need for toxic copper cocatalysts [13,14]. Notable pharmaceutical applications include the synthesis of Food and Drug Administration (FDA)-approved drugs such as Terbinafine, Ponatinib, and Tazarotene, highlighting the industrial relevance of copper-free conditions [15]. Examples of some FDA-approved medicines are given in Table 1 [7].

Similarly, the Heck reaction has found extensive application in pharmaceutical synthesis, with recent advances focusing on greener methodologies and improved regioselectivity for the preparation of trisubstituted alkenes as versatile pharmaceutical intermediates [16]. Modern approaches to the Heck reaction emphasize sustainable protocols that address both environmental concerns and the need for high-performance catalysts capable of operating under mild conditions [9].

However, despite the widespread industrial application of these palladium-catalyzed reactions, the pharmaceutical industry faces stringent regulatory challenges regarding metal contamination in final products. The quantification of catalyst loading in terms of parts per million (ppm) has gained significant attention in pharmaceutical applications, with regulatory requirements demanding palladium contents below 10 ppm for oral medicines according to European Medicines Agency guidelines [17]. Recent comprehensive reviews of palladium-catalyzed cross-coupling reactions emphasize the critical importance of achieving ultra-low metal contamination levels, particularly for active pharmaceutical ingredients (APIs) [18].

In medicine production, a crucial parameter is the absence or minimal content of the catalyst or cocatalyst (such as copper in the Sonogashira reaction) in the resulting product due to its adverse effects [19]. Homogeneous catalysis usually requires different steps for the purification of the target product from catalyst residues, which increases the cost of the resulting medicine and does not always demonstrate good results. Recent developments in palladium extraction methodologies have addressed these challenges through advanced scavenging technologies, including functionalized silicas and fixed-bed adsorption processes [17]. Lower residual catalyst concentration in the reaction product can be achieved by using heterogeneous catalysis [20].

Moreover, heterogeneous polymer-supported catalysts pave the way toward lower residual metal amounts in reaction products due to metal encapsulation and prevention of its leaching into the reaction media [21]. However, some polymers might degrade and age with time [22,23]. Contemporary research has focused on developing more stable and recyclable catalyst systems, with palladium nanoparticles emerging as a promising solution that combines the advantages of homogeneous and heterogeneous catalysis [24,25].

Compared to homogeneous catalysis, heterogeneous catalysis provides easy separation, recovery, and recycling of the catalyst [26]. Recent innovations in heterogeneous catalyst design have emphasized sustainable approaches, including the use of renewable supports, magnetic nanoparticle systems for easy separation, and environmentally benign reaction conditions [27]. The heterogeneous catalyst should be simple to prepare and use to provide many catalytic cycles [28]. Drawbacks of heterogeneous catalysis include lower yield and selectivity, as well as special procedures required for preparing heterogeneous catalysts [29].

The choice of support material plays a critical role in determining the overall performance of heterogeneous catalysts, with various options including carbon materials, metal oxides, zeolites, and organic polymers being extensively investigated. Among these diverse support systems, polysiloxane-supported catalysts represent a particularly promising class of heterogeneous systems due to their outstanding film-forming properties, thermal stability, and biocompatibility [1]. Unlike conventional rigid supports, polysiloxanes offer unique advantages including excellent processability, chemical inertness, and the ability to form flexible catalytic membranes. Recent studies have demonstrated the successful immobilization of palladium complexes on polysiloxane matrices, leading to recyclable catalysts with excellent performance in cross-coupling reactions [30,31].

Consequently, the development of high-performance, versatile heterogeneous Pd-containing polymer macrocatalysts stable for prolonged periods with simple preparation procedures opens opportunities for wide-ranging practical applications in different fields. The integration of green chemistry principles with advanced catalyst design has led to the development of sustainable catalytic systems that address both environmental and economic concerns [32]. Earlier, we studied the activity of palladium(II)-containing polysiloxanes (Pd-PDMS) in the Suzuki reaction; the catalyst demonstrated good yield (80%), no yield decrease for 3 catalytic cycles, and ultra-low levels of Pd in the target product [20]. Inspired by these promising results, we investigated the catalytic activity of Pd-PDMS in amine-, copper-, and phosphine-free Sonogashira reactions and amine- and phosphine-free Heck reactions due to their extensive use in synthesizing important and vital drugs (Table 1) and the necessity to create sustainable catalysts.

## 2. Materials and Methods

### 2.1. Materials

Phenylboronic acid (95%), 4-bromotoluene (98%), 4-iodoanisole (98%), phenylacetylene (98%), styrene containing 3,5-di-*tert*-butylcatechol as an inhibitor were purchased from Merck KGaA (St Louis, MO, USA). Poly((3-azidopropyl)methylsiloxane-*co*-dimethylsiloxane) (N_3_-PDMS) bearing 25 mol.% of (3-azidopropyl)methylsiloxane groups was obtained in accordance with the ref. [33]. The number-average molecular weight of N_3_-PDMS is equal to *M_n_* = 14,600 (dispersity *Đ* = 1.50). The Pd(II)-*C*,*N*-cyclometalated complex was synthesized and grafted to N_3_-PDMS in accordance with the previously published procedure [34].

### 2.2. Methods

Nuclear magnetic resonance (NMR) spectroscopy was carried out on the Bruker Avance III 400 NMR spectrometer (Bruker, Billerica, MA, USA) in CD_3_Cl and CD_3_OD at room temperature (RT) and 40 °C (at 400 MHz for ^1^H, 100 MHz for ^13^C). Chemical shifts in signals are reported in *δ*-values [ppm], relative to residual signals of solvents peaks for CDCl_3_: *δ* = 7.28 (^1^H), 77.2 (^13^C); for CD_3_OD: *δ* = 4.78 and 3.31 (^1^H), 49.2 (^13^C).

X-ray photoelectron spectroscopy (XPS) was performed on the Escalab 250Xi photoelectron spectrometer (Thermo Scientific, Waltham, MA, USA) with AlK_α_ radiation (photon energy 1486.6 eV). Spectra were recorded in the constant pass energy mode at 100 eV for survey spectrum and 50 eV for element core level spectrum, using XPS spot size 650 μm. A total energy resolution was approximately 0.3 eV. Investigations were carried out at RT in an ultrahigh vacuum of the order of 10^–9^ mbar. An ion-electronic charge compensation system was used to neutralize the charge of the samples during XPS measurements.

Raman spectra were obtained in backscattering geometry at the Raman spectrometer Senterra (Bruker, Bremen, Germany) equipped with 532 nm wavelength solid-state laser (the corresponding energy is 2.33 eV). The laser power under × 20 (numerical aperture = 0.4) objective was approximately 66 µW (*λ* = 532 nm). The diffraction grading was 400 lines·mm^–1^, and the aperture was 25 × 1000 µm. The spectra were recorded for 180 s with 3 repetitions.

Scanning electron microscopy (SEM) images of samples’ surfaces were obtained at RT on a Zeiss Auriga Crossbeam system (Carl Zeiss, Oberkochen, Germany) with an accelerating voltage of 20 kV and working distance from 9 to 12 mm.

ICP-AES. ICP-AES analysis was conducted on a Shimadzu ICPE-9000 spectrometer (Shimadzu, Kyoto, Japan). The palladium content was determined at λ = 367.47 nm. Prior to the analysis, the samples were refluxed in a mixture of 5.0 mL of hydrochloric acid and nitric acid (3:1, *v*/*v*). After cooling, the samples were quantitatively transferred to a volumetric flask, adjusted with a solution of 0.1 M HNO_3_ to 50 mL. Standard samples of analyzed elements were prepared from a multi-component standard (Merck, St. Louis, MO, USA) in 0.1 M HNO_3_ for a calibration curve (0.001–10 mg·dm^–3^).

Density functional theory (DFT) calculations. To simulate local binding of Pd-PDMS to the surface of carbon fibers of carbon paper, quantum chemical modeling within the DFT approach was carried out using Gaussian 09W Rev. C.01 (Gaussian Inc., Wallingford, CT, USA) [35]. To investigate structural peculiarities, the long-range dispersion corrected wB97XD exchange correlation functional was chosen [36]. Due to the fairly large number of atoms (220 atoms) in the investigated model, the compact double-zeta Pople-type 3-21G basis set was used [37,38]. Previously, this approach was used to study the interaction between non-substituted or pyrrolidine-substituted fullerene and porphyrins [39]. To model a part of the carbon fiber surface, a polyaromatic hydrocarbon (PAH) with 27 planar hexagonal rings was chosen. Hydrogen atoms were used to complete bonds to avoid dangling bonds. The structures of Pd-PDMS coordinated to PAH were optimized until the standard criteria for maximal forces and maximal atomic displacements as well as root-mean-square (RMS) forces and RMS atomic displacements were met. The stability of optimized geometries was confirmed by the absence of imaginary frequencies. The visualization of structures was performed with the aid of GaussView 5.0.9 software (Gaussian Inc., Wallingford, CT, USA) and the Origin 9.0 software (OriginLab Co.; Northampton, MA, USA).

### 2.3. Pd-PDMS Application on the Carbon Support

A required amount of Pd-PDMS was dissolved in 75 μL of CH_2_Cl_2_. As a carbon support, carbon paper (CP) was chosen. Raman spectrum of the initial CP is presented in Figure S2 of the Supplementary Materials. A piece 3 × 15 mm of CP was soaked in *i*-PrOH for 10 min and then sonicated for 3 min in an ultrasonic bath. The piece of CP was dried at 100 °C for 1 h. The pre-prepared solution of Pd-PDMS in CH_2_Cl_2_ was carefully applied by drop-casting on the CP in a stream of hot air to enable a quick evaporation of the solvent and good distribution of Pd-PDMS on the carbon support. The obtained catalytic membrane was dried at 50 °C for 1 h.

### 2.4. Catalytic Reactions

#### 2.4.1. Sonogashira Reaction with Pd-PDMS Catalytic Membrane

4-Iodoanisole (1.0 × 10^–4^ mol, 1.0 equiv), phenylacetylene (1.5 × 10^–4^ mol, 1.5 equiv) and K_2_CO_3_ (2.5 × 10^–4^ mol, 2.5 equiv) were weighted in a 2 mL vial, and 0.8 mL of CD_3_OD was added. A pre-prepared catalytic membrane containing 2.1 mg of Pd-PDMS was placed in the vial. The catalyst loading was 0.1 mol.% (calculated on Pd metal). A gentle stream of argon was blown to the vial. The vial was tightly sealed and weighted. The reaction was carried out at 55 °C for 8 h under a constant stirring. The vial was weighed after completion of the reaction; no weight loss was detected. Afterwards 0.6 mL of the reaction mixture was placed an NMR tube and ^1^H NMR spectrum was registered in order to calculate the yield of 1-methoxy-4-(phenylethynyl)benzene. The yield of the target product was calculated by comparing integrated intensities of peaks corresponding to the methoxy group of initial 4-iodoanisole and the target product. Yield of 1-methoxy-4-(phenylethynyl)benzene was 90%.

#### 2.4.2. Control of the Stability of 4-Iodoanisole in the Sonogashira Conditions Without Phenylacetylene

4-Iodoanisole (1.0 × 10^–4^ mol, 1.0 equiv) and K_2_CO_3_ (2.5 × 10^–4^ mol, 2.5 equiv) were weighted in a 2 mL vial, and 0.8 mL of CD_3_OD was added. A pre-prepared catalytic membrane containing 2.1 mg of Pd-PDMS was placed in the vial. A gentle stream of argon was blown to the vial. The vial was tightly sealed and weighted. The reaction was carried out at 55 °C for 8 h under a constant stirring. The vial was weighed after completion of the reaction; no weight loss was detected. An aliquot of 0.6 mL of the sample was analyzed by ^1^H NMR.

#### 2.4.3. Heck Reaction with Pd-PDMS Catalytic Membrane

4-Bromotoluene (1.0 × 10^–4^ mol, 1.0 equiv), styrene (1.5 × 10^–4^ mol, 1.5 equiv) and K_2_CO_3_ (2.5 × 10^–4^ mol, 2.5 equiv) were weighted in a 2 mL vial, and 0.8 mL of CD_3_OD was added. A pre-prepared catalytic membrane containing 1.7 mg of Pd-PDMS was placed in the vial. The catalyst loading is 0.1 mol.% (calculated on Pd metal). A gentle stream of argon was blown to the vial. The vial was tightly sealed and weighted. The reaction was carried out at 90 °C for 24 h under a constant stirring. The vial was weighed after completion of the reaction; no weight loss was detected. Afterwards 0.6 mL of the reaction mixture was placed an NMR tube and ^1^H NMR spectrum was registered in order to calculate the yield of 1-methyl-4-styrylbenzene. The yield of the target product was calculated by comparing integrated intensities of peaks corresponding to the methyl group of initial 4-bromotoluene and the target product. Yield of 1-methyl-4-styrylbenzene was 80% (*E*/*Z* ratio > 99:1).

#### 2.4.4. Control Experiment with Styrene in the Heck Reaction Conditions (Checking Styrene Polymerization)

Styrene (1.0 × 10^–4^ mol, 1.0 equiv) and K_2_CO_3_ (2.5 × 10^–4^ mol, 2.5 equiv) were weighted in a 2 mL vial, and 0.8 mL of CD_3_OD was added. A pre-prepared catalytic membrane containing 1.7 mg of Pd-PDMS was placed in the vial. A gentle stream of argon was blown to the vial. The vial was tightly sealed and weighted. The reaction was carried out at 90 °C for 24 h under a constant stirring. The vial was weighed after completion of the reaction; no weight loss was detected. An aliquot of 0.6 mL of the sample was analyzed by diffusion ordered NMR spectroscopy using bipolar pulse-gradient pulse (stebpgp) sequence with a pulse width 8.5 s and relaxation delay 2.0 s.

## 3. Results and Discussion

### 3.1. Catalytic Membrane Fabrication and Characterisation by SEM and XPS

**Figure 1:**
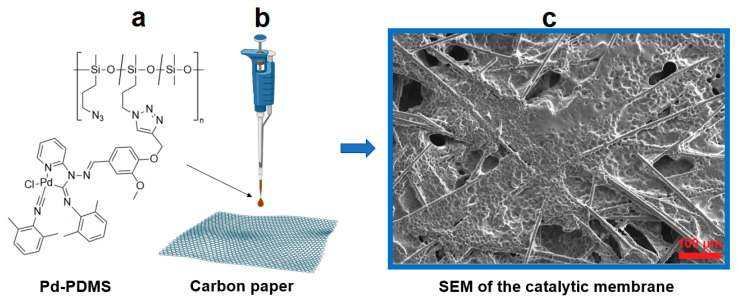
Structure of Pd-PDMS (**a**); scheme of catalytic membrane Pd-PDMS preparation (**b**); SEM image of the Pd-PDMS-containing catalytic membrane (**c**).

Palladium(II)-containing polysiloxane (Pd-PDMS) for catalytic membrane preparation was fabricated using N_3_-PDMS functionalized with the palladium(II) *C*,*N*-cyclometalated complex (structure of Pd-PDMS is shown in Figure 1; scheme of Pd-PDMS synthesis is presented in Figure S3). Inspired by our previous promising results, we analyzed the stability and catalytic performance of Pd-PDMS after one year of preservation in aerial atmosphere at RT.

First, we checked the solubility of Pd-PDMS. As before, Pd-PDMS is soluble in CHCl_3_ and CH_2_Cl_2_ but insoluble in alcohols. Second, we confirmed the unchanged structure of Pd-PDMS by ^1^H NMR spectroscopy. The ^1^H NMR spectra of freshly prepared Pd-PDMS and the same sample stored for one year are presented in Figure S4. Third, for catalytic membrane preparation, Pd-PDMS was dissolved in CH_2_Cl_2_ to provide quick solvent evaporation from the carbon support, the solution was drop-cast on CP, and the paper was dried at 50 °C for 1 h. The scheme of catalytic membrane preparation is given in Figure 1. A scanning electron microscopy (SEM) image of the obtained catalytic membrane is also shown in Figure 1.

In SEM images of the catalytic membrane, carbon fibers with thickness of 4–6 μm are visible. Each fiber is thoroughly covered with a layer of Pd-PDMS. Pd-PDMS forms a slightly porous covering on the carbon fibers of carbon paper (CP), which might be advantageous for the catalytic performance of the resulting catalytic membrane. More SEM images of the initial CP and the catalytic membrane are provided in Figure S6.

**Figure 2:**
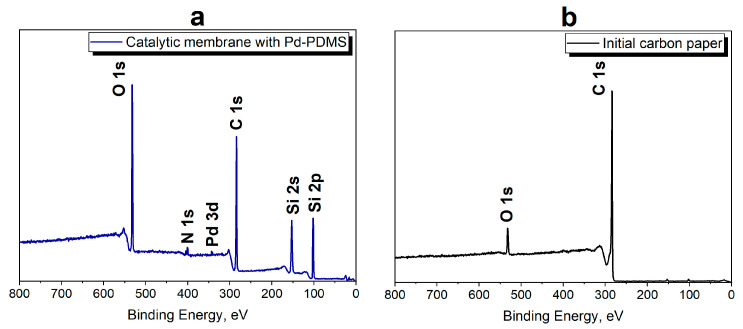
XPS survey spectra of the catalytic membrane with Pd-PDMS (**a**) and the initial CP (**b**).

The presence of Pd-PDMS on the carbon support was confirmed by XPS. XPS survey spectra of the catalytic membrane with Pd-PDMS and the initial CP are presented in Figure 2. XPS core level spectra of Si 2p and Pd 3d are given in Figure S7.

In the XPS spectra of initial CP, there are bands with binding energy (BE) values of 284.5 and 532.3 eV corresponding to C 1s and O 1s, respectively. The presence of C 1s is due to Csp^2^ structures in the carbon support (see Raman spectra in Figure S2), while oxygen-containing groups are formed during CP fabrication [40]. After treatment of CP with Pd-PDMS, new bands with BE of 337.6 eV (Pd 3d) and 102.1 eV (Si 2p) appear. The Pd 3d BE value is in agreement with data reported earlier for Pd(II)-*C*,*N*-cyclometalated complex in ref. [20]. The BEs of Si 2p and Si 2s are close to those of a similar polymer-metal complex containing Pt(II)-*C*,*N*-cyclometalated groups [34] and other metal-containing (poly)siloxanes [41]. Thus, the presence of Pd-PDMS in the catalytic membrane is confirmed.

**Figure 3:**
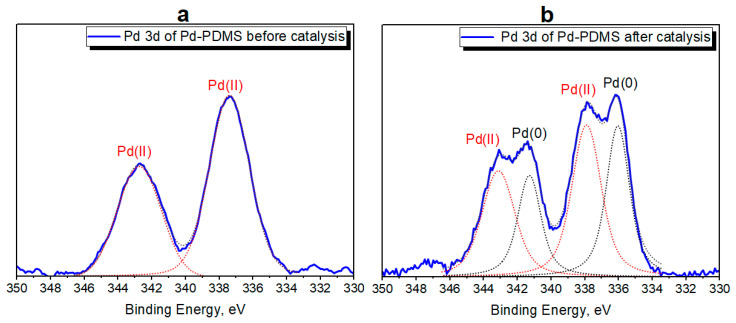
High-resolution Pd 3d XPS spectra of Pd-PDMS catalytic membrane before catalysis (**a**) and after catalysis (**b**).

In order to evaluate the changes in Pd-catalytic sites of Pd-PDMS, we acquired Pd 3d core level spectra before and after catalysis. As an example, the Suzuki reaction was chosen. Deconvolution of Pd 3d core level spectra for Pd-PDMS-containing catalytic membrane was performed in accordance with refs. [42,43] (Figure 3). Background subtraction was carried out using the Tougaard method. The XPS peaks usually demonstrate Lorentzian contour, but the presence of instrumental and other factors leads to a Gaussian contribution. Thus, the mixed Gaussian–Lorentzian function was used for the Pd 3d core level fitting [44].

In high-resolution Pd 3d XPS spectra of Pd-PDMS before catalysis Pd 3d_3/2_ and Pd 3d_5/2_ demonstrates value of BE at 342.7 and 337.4 eV, which corresponds Pd(II) species. After the catalytic cycles, in XPS spectra of Pd-PDMS, two Pd species are presented corresponding Pd(II) species and Pd(0). The BE of Pd 3d_3/2_ and Pd 3d_5/2_ for Pd(II) is equal for 342.9 and 337.7 eV, which is close to the initial Pd(II) BE values and indicate unchanged Pd environment for the remaining active cites. The relative intensity ratio of the two species (Pd(II) and Pd(0)) is approximately 50:50, which indicates the presence of the initial form of the catalyst alongside with a Pd(0) form. Therefore, we confirmed the unchanged structure of the remaining active sites in Pd-PDMS after catalysis.

### 3.2. Catalytic Performance

**Figure 4:**
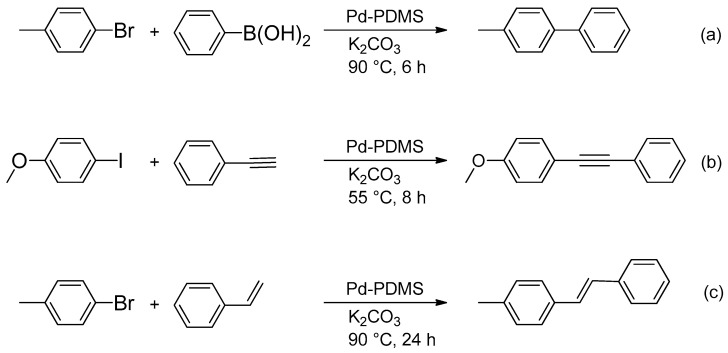
Reactions catalyzed by the Pd-PDMS catalytic membrane: Suzuki coupling (**a**), Sonogashira coupling (**b**), Heck coupling (**c**).

In our earlier study [20], we reported that freshly prepared palladium-containing polysiloxane exhibits catalytic activity in the Suzuki reaction. In this work, we found the important phenomenon that Pd-PDMS retains its catalytic activity even when stored in air for one year. The yield of the product of the Suzuki reaction between 4-bromotoluene and phenylboronic acid in a mixture of CD_3_OD:D_2_O in *v*/*v* ratio (4:1) in the presence of the Pd-PDMS catalytic membrane is the same as for the fresh catalyst (80%), which is a highly promising result for practical application of the developed heterogeneous catalyst. No yield decrease was observed in the two following catalytic cycles (80% in each cycle). The reaction scheme is shown in Figure 4a. ^1^H NMR spectra of the catalytic reactions are presented in Figure S8.

After confirmation of Pd-PDMS stability (see Section 3.2 “Stability of Pd-PDMS” in SI; ^1^H NMR Pd-PDMS is in Figure S4, IR spectrum is in Figure S5) and maintenance of its catalytic activity in the Suzuki reaction, we proceeded to investigate other cross-coupling reactions, namely copper-free Sonogashira and Heck cross-couplings. Usually, palladium-catalyzed Sonogashira cross-coupling is performed in the presence of copper cocatalyst [45]. As the cocatalyst is toxic, it is highly desirable to conduct the Sonogashira reaction in the absence of copper [13,46].

We carried out the copper-free Sonogashira reaction catalyzed by the Pd-PDMS catalytic membrane between 4-iodoanisole and phenylacetylene, which were taken in a molar ratio 1:1.5 in the presence of K_2_CO_3_ as base in CD_3_OD in a tightly sealed vial. We did not use amines as base or phosphine ligands in the reaction. The reaction scheme is shown in Figure 4b. After the reaction was carried out for 4 h, the yield of target 1-methoxy-4-(phenylethynyl)benzene, calculated from ^1^H NMR data, was 45%. Increasing the reaction time to 8 h allowed enlargement of the yield to 90%. Further reaction (up to 24 h) does not significantly influence the yield. Therefore, we chose 8 h as the optimal reaction time.

The reaction temperature was chosen in accordance with ref. [47] for polymer-supported Pd(II) catalyst, where good yield (approximately 90%) was achieved at 70 °C. We were able to decrease the temperature to 55 °C to achieve 90% yield of 4-methoxytolane. Therefore, the macrocatalyst demonstrated good activity in the absence of copper cocatalyst and toxic amines and phosphine ligands. As we established conditions for successful reaction, the reusability and recyclability of the Pd-PDMS catalytic membrane were tested in the Sonogashira reaction. After each catalytic cycle, the Pd-PDMS catalytic membrane was removed from the vial, washed with CH_3_OH, dried, and used again. The Sonogashira reaction with the same Pd-PDMS catalytic membrane was performed for two more cycles. No yield decrease was detected (90% in each cycle). ^1^H NMR spectra of the catalytic reactions are presented in Figure S9. We also tested the possibility of Pd-catalyzed side C–O cross-coupling reaction between 4-iodoanisole and CD_3_OD in the absence of phenylacetylene. In a blank experiment, no side reactions were observed.

Considering the success of Suzuki and Sonogashira reactions, we decided to inspect the catalytic activity of the Pd-PDMS catalytic membrane in the Heck reaction. The Heck reaction was performed between 4-bromotoluene and styrene in a molar ratio 1:1.5 in the presence of K_2_CO_3_ as base. The reaction scheme is shown in Figure 4c.

For the Heck reaction, we used styrene containing 3,5-di-tert-butylcatechol to prevent its possible thermal polymerization [48,49]. It is known [50,51] that the Heck reaction requires relatively high temperature and long reaction time. Thus, the reaction was conducted at 90 °C. Before performing the Heck reaction, we tested the possibility of styrene polymerization in the presence of the Pd-PDMS complex at 90 °C in CD_3_OD. The experiment was conducted for 24 h. No visual presence of polystyrene in the form of colorless substance or powder was detected in the test reaction. The test reaction was analyzed by ^1^H NMR and diffusion-ordered NMR spectroscopy and compared with initial styrene (Figure S10). NMR confirmed the absence of styrene polymerization. This might be due to the presence of inhibitor in styrene, which was intentionally preserved in the substance.

Regarding the catalytic activity of Pd-PDMS in the Heck reaction, 24 h and 90 °C were found to be good conditions to achieve 80% yield of the desired 1-methyl-4-styrylbenzene. After the reaction, we separated the catalyst and used it again after washing. The experiment showed that the catalytic activity of the membrane did not change, which proves the possibility of recycling this catalyst under Heck reaction conditions (Figure S11).

Therefore, applying Pd-PDMS allowed us to perform Suzuki, copper-free Sonogashira, and Heck reactions. For the first two reactions, we performed 3 catalytic cycles without decreasing yields of target products, and for the Heck reaction, 2 catalytic cycles. We summarized the obtained data in Table S1. After each catalytic cycle, the Pd-PDMS catalytic membrane was removed from the reaction mixture, cleaned, and used again.

Due to the importance of low palladium levels in pharmaceutically active substances, we determined the concentration of palladium after the reaction by ICP-AES method. The palladium concentration was 22 and 167 ppb for products of Sonogashira and Heck couplings, respectively, which is significantly lower than the palladium level required by the European Medicines Agency (10 ppm) [17]. In our previous study [20], we reported that the palladium content in product of Suzuki coupling catalyzed by freshly prepared Pd-PDMS is 22 ppb. In this study, we conducted the Suzuki reaction with Pd-PDMS preserved in air for a year and obtained similar results on the palladium concentration in the product of Suzuki reaction (See detail in chapter S6 “Catalytic performance”). This indicates an almost complete absence of palladium leaching from the catalytic membrane and the potential applicability of Pd-PDMS in the industrial synthesis of various active pharmaceutical ingredients (APIs) [17].

### 3.3. DFT Calculations

**Figure 5:**
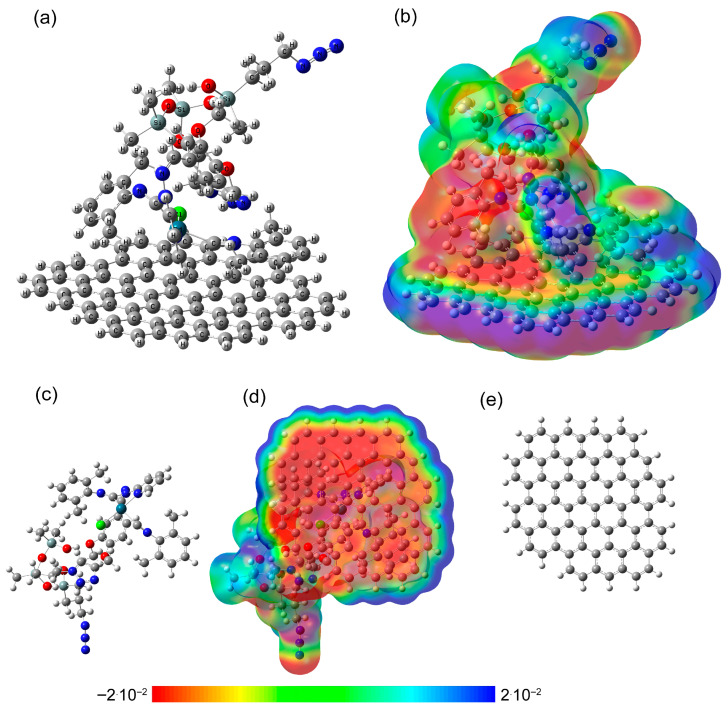
Optimized geometry of Pd-PDMS complex coordinated to PAH (**a**) and corresponding ESP map (**b**). Bottom views of the optimized geometry showing (**d**) the Pd-PDMS complex coordinated to PAH; (**c**) the Pd-PDMS complex part only, and (**e**) the PAH part only. ESP map values are given in eV.

To estimate local interaction of Pd-PDMS and the surface of carbon fibers of carbon paper, DFT calculations were performed. As a model compound simulating a part of the carbon fiber surface, polyaromatic hydrocarbon (PAH) with 27 planar hexagonal rings was chosen. Similar approaches were used in previous studies, where dimers and trimers of polycyclic aromatic hydrocarbons were used as models of graphene bilayers and trilayers [52] as well as to model interaction with multi-walled carbon nanotubes outer layer [53]. The optimized geometry of Pd-PDMS complex coordinated to PAH with corresponding electrostatic potential (ESP) maps is depicted in Figure 5.

In the choice of the planar PAH, we took into account the curvature of carbon fibers, which is smaller than the curvature of conventional multi-walled carbon nanotubes [54]. Moreover, the surface area was chosen in such a way that, on the one hand, there would be no difficulties in approaching, and on the other hand, to minimize possible boundary effects. As a part of the polysiloxane chain, a unit bearing a 3-azidopropyl group and a unit with Pd(II)-*C*,*N*-cyclometalated complex were chosen.

At the first step, the initial structures of PAH and Pd-PDMS fragments were optimized. The geometry of obtained structures and corresponding ESP maps are presented in Figure S12. In the optimized structure of Pd-PDMS, two xylyl rings have different environments. One of the xylyl groups is influenced by the electronegative nitrogen of the triazole ring. Additionally, the relative flexibility of C_Ar_–N and Pd–C bonds leads to the orientation of two protons of one of the methyl groups of the xylyl ring toward the triazole ring. The orientation of the second methyl group depends on the position of the methyl group of the polysiloxane chain and the C_Ar_–N bond alongside the presence of the electronegative chlorine atom.

After coordination of the Pd-PDMS fragment to the PAH surface, one of the xylyl rings has a parallel offset orientation to the PAH surface [55]. The shortest predicted distance between PAH and the xylyl ring is approximately 3.26 Å, while the shortest distance between one of the protons of the methyl group of the xylyl ring and PAH is approximately 2.41 Å. The other xylyl ring is oriented with individual hydrogens of the methyl group, forming a region of positive charge, toward the electronegative region of PAH (Figure 5 and Figure S12). The shortest distances from them to the nearest carbon atoms in PAH are within 2.65–2.71 Å. Moreover, at this location, a change in carbon-carbon bond lengths in PAH is observed over the largest area (Figure S13).

Thus, specific coordination interaction between the palladium complex and carbon support surface was found and analyzed. The increased interaction between Pd(II)-*C*,*N*-cyclometalated complex and PAH surface explains the effectiveness of the carbon support for the palladium–polysiloxane macrocatalyst design.

## 4. Conclusions

This work presents a carbon-supported Pd-PDMS system that addresses critical challenges in pharmaceutical catalysis: metal contamination, catalyst recyclability, and elimination of toxic cocatalysts. The exceptional year-long storage stability at ambient conditions represents a significant advance for polymer-supported metal catalysts. Our Pd-PDMS system maintains full catalytic activity after one year of aerial storage, with Suzuki reaction yields remaining at 80% for both fresh and aged catalyst, addressing a fundamental barrier to industrial adoption.

The catalyst demonstrates comprehensive cross-coupling versatility by efficiently promoting three pharmaceutical-relevant reactions. Suzuki reaction yields reached 80%, copper-free Sonogashira achieved a yield 90% at 55 °C, and Heck reaction afforded a yield 80% at 95 °C. The copper-free Sonogashira conditions eliminate toxic copper cocatalysts, phosphine ligands, and organic bases while operating under milder conditions.

Ultra-low metal contamination represents the most significant achievement from a regulatory perspective. Palladium concentrations of 22 ppb for Sonogashira and Suzuki products, and 167 ppb for Heck products represent a 60–450-fold improvement over European Medicines Agency requirements of 10 ppm [17], potentially eliminating costly downstream metal scavenging steps. The catalyst exhibits excellent recyclability without activity loss over multiple cycles, with consistent yields maintained throughout. The simple washing and drying protocol between cycles supports industrial implementation requirements.

DFT calculations reveal specific coordination interactions between the palladium complex and carbon support surface, with the xylyl ring orienting parallel to the carbon surface at 3.26 Å distance and additional CH–π interactions at 2.41–2.71 Å. This transforms catalyst design from empirical optimization to deliberate engineering based on molecular interactions.

The convergence of exceptional stability, pharmaceutical-grade metal contamination control, and multi-reaction versatility establishes this Pd-PDMS system as a significant advancement for sustainable cross-coupling catalysis. This work demonstrates that properly designed polymer-supported catalysts can meet pharmaceutical requirements while providing economic and environmental benefits through reduced metal waste and simplified purification procedures.

## Figures and Tables

**Table 1:** Some of the medicines synthesized using Suzuki, Sonogashira, and Heck reactions [7]. The structures of the listed medicines are presented in Figure S1 of the Supplementary Materials.

Medicine	Pharmacological Activity or Disease	Reaction
Selpercatinib	Lung and thyroid cancer	Suzuki reaction
Darolutamide	Prostate cancer	Suzuki reaction
Siponimod	Multiple sclerosis	Suzuki reaction
Tezacaftor	Cystic fibrosis	Sonogashira reaction
Ribociclib	Breast cancer	Sonogashira reaction
Letermovir	Antiviral drug	Heck reaction
Palbociclib	Anticancer activity	Heck reaction

## Data Availability

The data supporting this article have been included as a part of the Supplementary Materials.

## Supplementary Materials

The following supporting information can be downloaded at: https://www.mdpi.com/article/10.3390/polym17223066/s1, Figure S1: Some examples of medicines, which can be synthesized using C–C cross-couplings; Figure S2: Raman spectrum of the initial carbon paper; Figure S3: Synthesis of Pd-PDMS; Figure S4: ^1^H NMR of freshly prepared Pd-PDMS (a) and after one year of preparation in aerial atmosphere at RT (b); Figure S5: IR spectrum of Pd-PDMS in the area of 550–450 cm^–1^ (a) and 100–600^–1^ (b); Figure S6: SEM images of the initial carbon paper (a), Pd-PDMS catalytic membrane before catalysis (b), Pd-PDMS catalytic membrane after catalysis (c); Figure S7: XPS core level spectra of Si 2p and Pd 3d; Figure S8: ^1^H NMR of Suzuki coupling; Figure S9: ^1^H NMR of Sonogashira coupling; Figure S10: DOSY initial styrene (a) and after control test in Heck coupling conditions (b); Figure S11: ^1^H NMR of Heck coupling; Figure S12: The geometry of Pd-PDMS (a) and PAH (b) and corresponding electrostatic potential maps; Figure S13: Bond lengths in PAH; Table S1: The summary of the catalytic performance of Pd-PDMS catalytic membrane; Table S2: TON and TOF for Pd-PDMS. Refs. [56,57,58,59,60,61] cited in Supplementary Materials.
