# Supplementary material for: Recyclable Palladium-Polysiloxane Catalyst with Ultra-Low Metal Leaching for Drug Synthesis
Source: Polymers (Basel). 2025 Nov 19;17(22):3066. doi: 10.3390/polym17223066 (PMC12656307; doi:10.3390/polym17223066)
Supplement: Supplementary file 1 [file polymers-17-03066-s001.zip › polymers-3936652-supplementary.pdf]

Supporting Information

Recyclable Palladium-Polysiloxane Catalyst with Ultra-Low Metal Leaching for Drug Synthesis

Ekaterina A. Golovenko, Polina P. Petrova, Dmitrii V. Pankin, Sergey V. Baykov, Vadim Yu. Kukushkin, Vadim P. Boyarskiy*, and Regina M. Islamova*

St. Petersburg State University, 7/9 Universitetskaya nab., St. Petersburg, 199034 Russia;

*Corresponding authors

E-mail address: v.boiarskii@spbu.ru (V.P.B.), r.islamova@spbu.ru (R.M.I.)

Contents

S1. Examples of synthetic capabilities of C–C cross-couplings S2
S2. Characterization of the initial carbon paper S3
S3. Pd-PDMS S3
S3.1. Synthesis of Pd-PDMS S3
S3.2. Stability of Pd-PDMS S4
S4. SEM images of Pd-PDMS catalytic membrane S6
S5. XPS data S6
S6. Catalytic performance S6
S6.1. Suzuki reaction S6
S6.2. Sonogashira reaction S8
S6.3. Heck reaction S8
S7. TON and TOF calculations S10
S8. DFT calculations S11

S1. Examples of synthetic capabilities of C–C cross-couplings

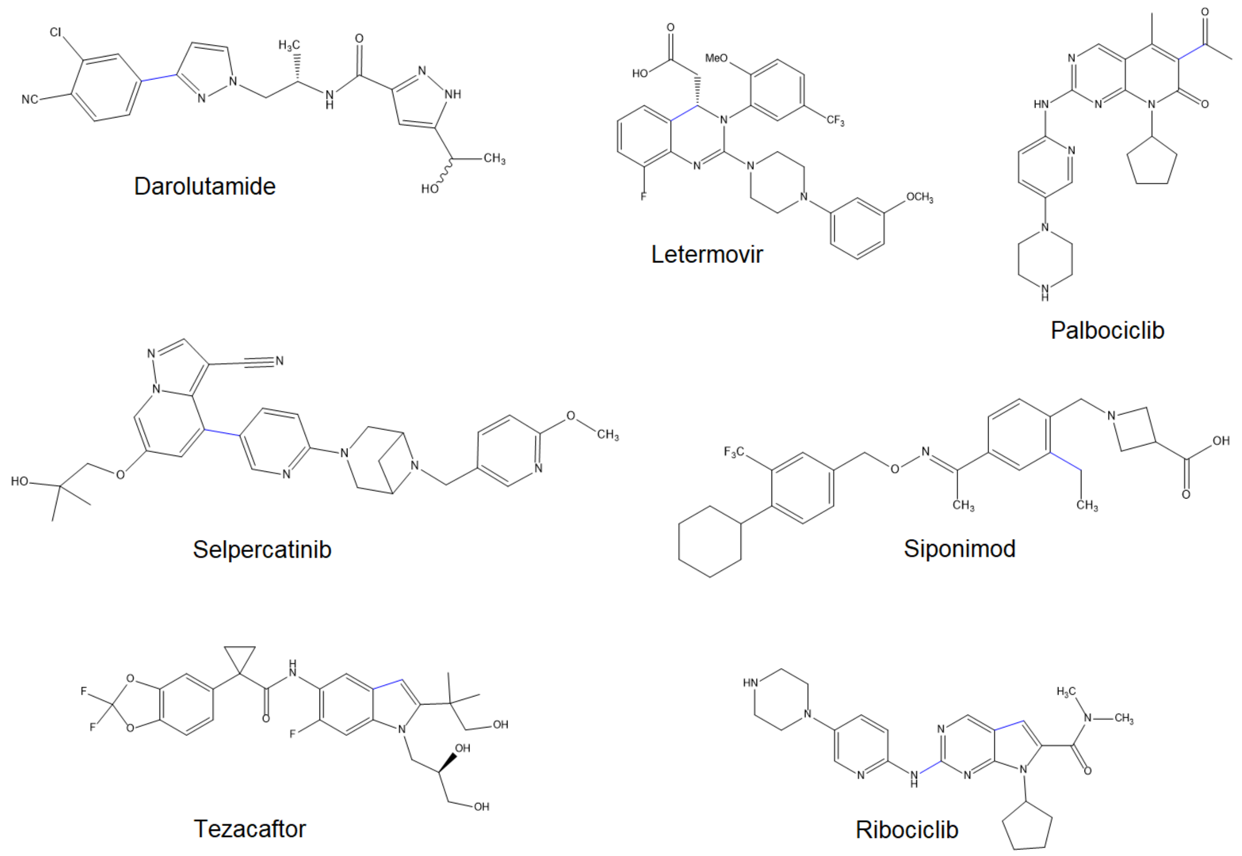

Figure S1. Some examples of medicines, which can be synthesized using C–C cross-couplings.

S2. Characterization of the initial carbon paper

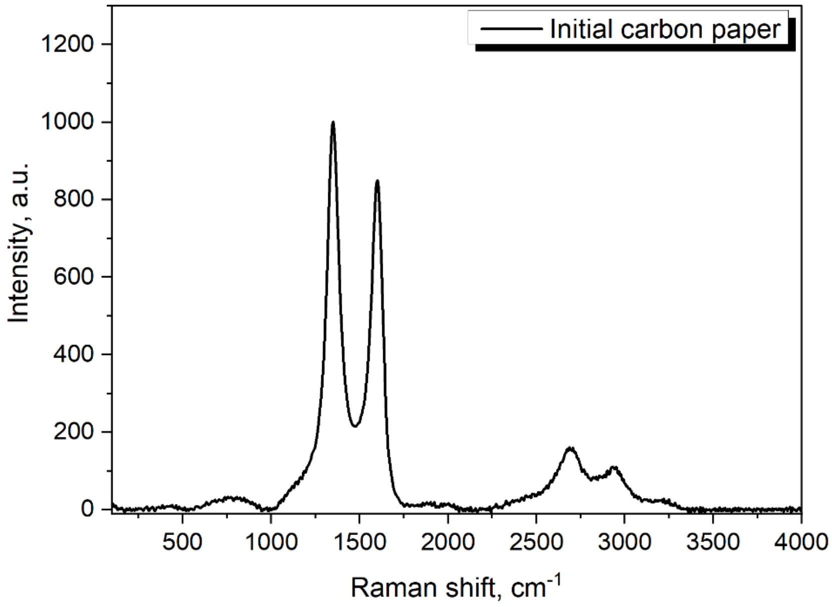

Figure S2. Raman spectrum of the initial carbon paper.

S3. Pd-PDMS

S3.1. Synthesis of Pd-PDMS

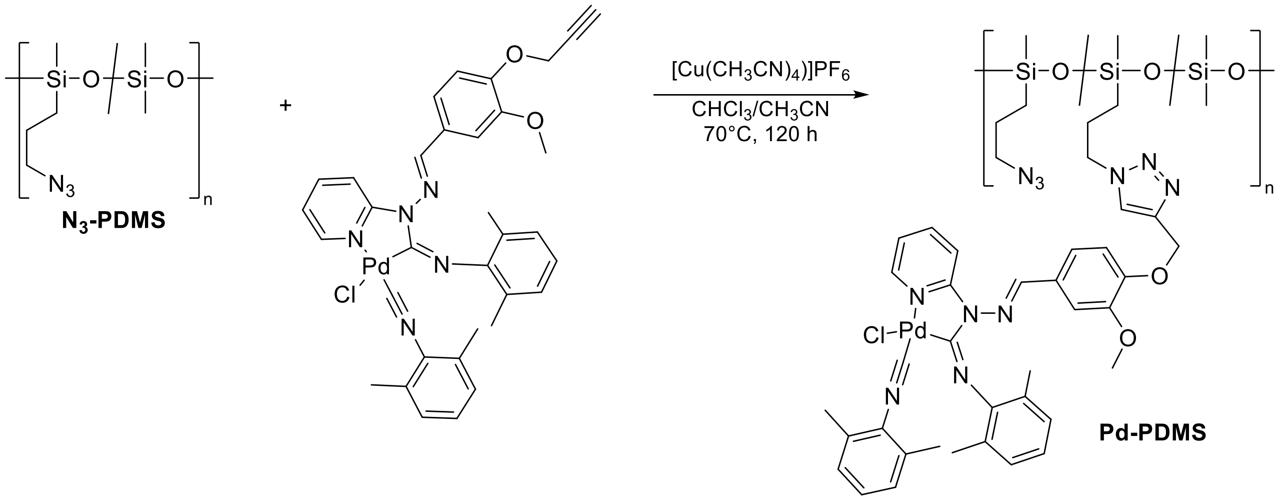

Figure S3. Synthesis of Pd-PDMS.

S3.2. Stability of Pd-PDMS

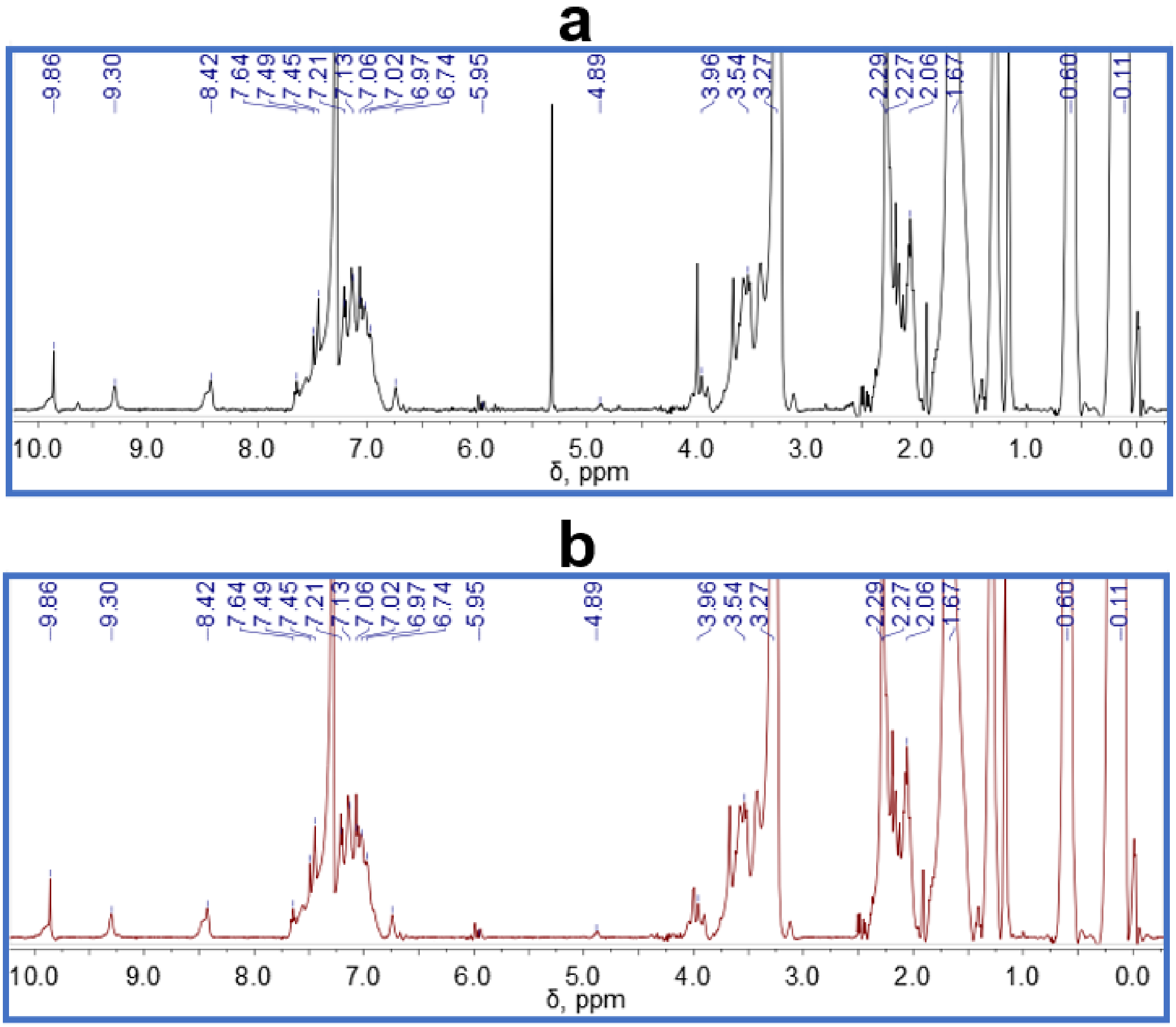

Figure S4. ^{1}H NMR of freshly prepared Pd-PDMS (a) and after one year of preparation in aerial atmosphere at RT (b).

Pd-PDMS ^{1}H NMR ($CDCl_3$-*d*, δ): 9.86 (br. s, *l*-H), 9.30 (br. m, *h*-H), 8.42 (br. s, *v*-H), 7.64 (br. t, *f*-H), 7.49 (br. s, *i*-H), 7.45 (br. d, *e*-H), 7.41 (br. m, *k*-H), 7.13 (br. m, *j*-H), 7.06 (br. m, *q*-H), 7.02 (br. m, *p*-H), 6.97 (br. m, *g*-H), 6.74 (br. m, *s*-H), 5.95 (br. m, *t*-H), 4.89 (m, *n*-H), 3.96 (s, *m*-H), 3.54 (br. m, $SiCH_2CH_2C\mathit{H_2}N$), 3.27 (br. m, high intensity, $SiCH_2CH_2C\mathit{H_2}N_3$), 2.29 (br. s, *r*-H), 2.27 (br. s, *u*-H), 2.06 (br. m, $SiCH_2C\mathit{H_2}CH_2N$), 1.67 (br. m, high intensity, $SiCH_2C\mathit{H_2}CH_2N_3$), 0.60 (br. m, $SiC\mathit{H_2}CH_2CH_2N$), 0.1 (br. s, high intensity, $SiC\mathit{H_3}$).

In order to additionally confirm the preservation of Pd-PDMS structure, IR-spectrum was obtained (**Figure S5**).

The IR absorbance spectrum was obtained at the FTIR spectrometer Nicolet 8700 (Thermo Fisher Scientific, Waltham, MA, USA) with deuterated triglicine sulphate detector (DTGS) with thermoelectric Peltier cooling using attenuated total reflection (ATR) accessory in the 520–4000 cm^{-1}. The ATR crystal was diamond. The spectral resolution was 4 cm^{-1}. The light source was SiC (Globar). The aperture size was 70 percent of maximum. Additionally, the IR absorbance spectrum was obtained using diffuse reflectance Infrared Fourier-transform spectroscopy (DRIFTS) technique in the 100-600 cm^{-1} with corresponding accessory. DTGS detector with polyethylene (PE) windows was used. In both cases the spectral resolution was 4 cm^{-1} The demonstrated spectra are the result of averaging by 100 scans.

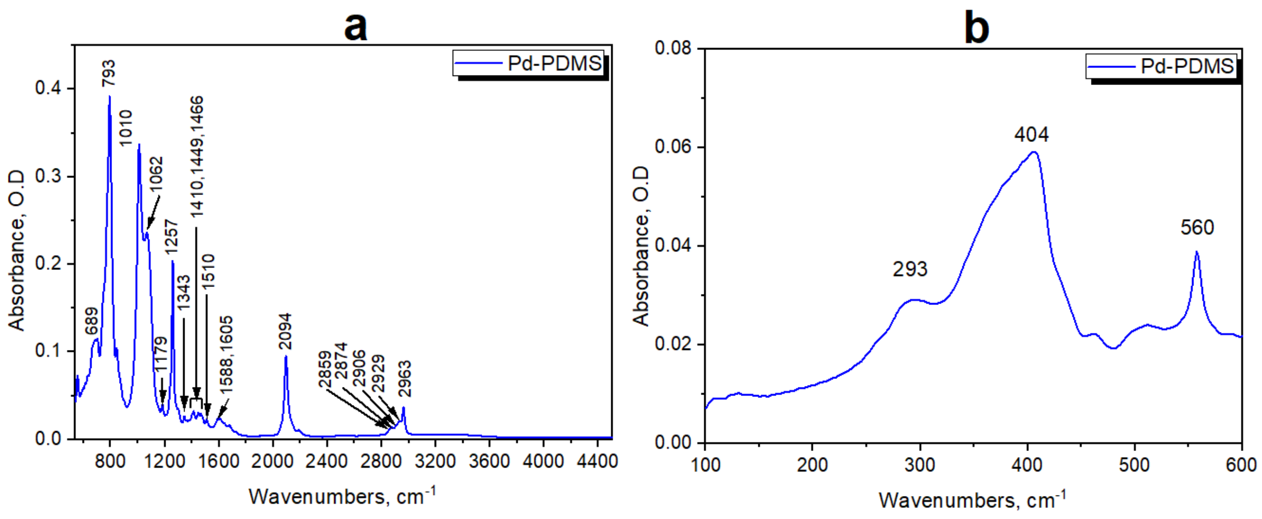

Figure S5. IR spectrum of Pd-PDMS in the area of 550–450 cm^{-1} (a) and 100–600^{-1} (b)

In the obtained IR spectrum (**Figure S5a**), a set of absorption peaks can be distinguished at 793, 1010, 1062, and 1257 cm^{-1}, which are located in the spectral regions typical for polysiloxanes. [1,2] The peak at 793 cm^{-1} is interpreted as antisymmetric rocking in CH_3 coupled with Si—C stretching vibrations, which is accompanied by symmetric CH_3 rocking vibrations at 689 cm^{-1}.[1] The peaks at 1010 and 1062 cm^{-1} are in the region where vibrations in the silicon-oxygen region may occur. [1,2] The peaks at 1010 and 1062 cm^{-1} are close in frequency to the symmetric and antisymmetric stretching vibrations in Si—O—Si chain of polysiloxanes. The peak at 1257 cm^{-1} is related to bending vibrations in the CH_3 group attached to the Si atom. Additionally, the IR absorption spectrum contains an asymmetric band with a maximum at 2963 cm^{-1}, which corresponds to antisymmetric vibrations in the CH_3 group. [1,2] This peak frequency is typical for similar vibrations in polysiloxanes. The asymmetry of the band arises due to additional poorly resolved contributions from symmetric and antisymmetric stretching of hydrogen vibrations in methylene groups (2856 and 2929 cm^{-1}), respectively, and symmetric stretching hydrogen vibrations in methyl groups in general (2874 cm^{-1}) and polysiloxanes in particular (2906 cm^{-1}).[1] The obtained spectrum also shows the presence of hydrogen vibrations in the region of bending hydrogen vibrations at 1400-1475 cm^{-1}, where the contribution comes not only from methyl and methylene groups, but also from xylil rings. Additionally, in the 1500–1600 cm^{-1} region, the contribution of heterogeneous C=N bonds can

possibly contribute. The manifestation of C—C, C—O, and C—N single bonds in this spectrum most likely appears as weak peaks at 1179 and 1343 cm^{-1}.

Considering Pd—N, Pd—C and Pd—Cl vibrations, presumably, they might appear in low frequency region (**Figure S5b**). The peak at 293 cm^{-1} is assigned for Pd—Cl stretching vibrations,[3] while peaks at 404 and 560 cm^{-1} might be associated with Pd–N and/or Pd–C stretching vibrations.

S4. SEM images of Pd-PDMS catalytic membrane

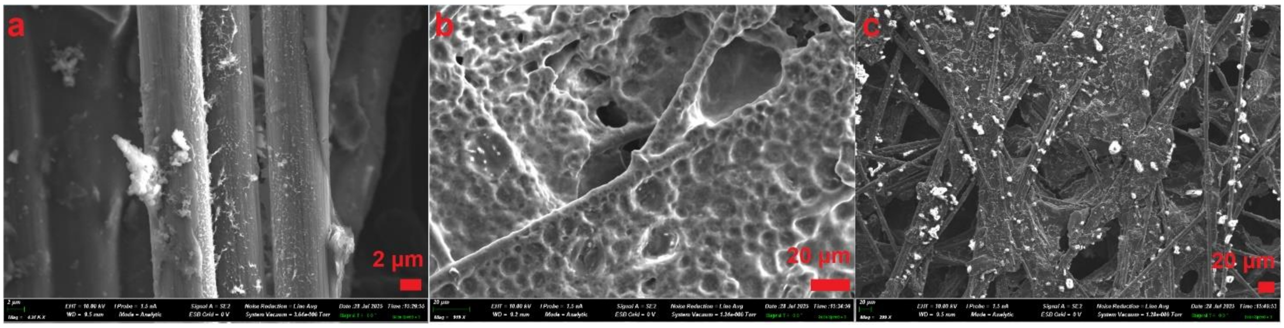

Figure S6. SEM images of the initial carbon paper (a), Pd-PDMS catalytic membrane before catalysis (b), Pd-PDMS catalytic membrane after catalysis (c).

S5. XPS data

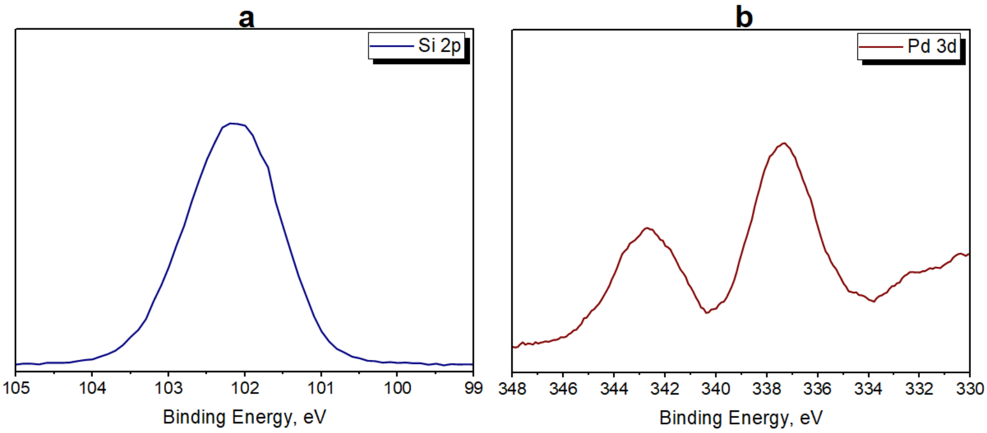

Figure S7. XPS core level spectra of Si 2p and Pd 3d

S6. Catalytic performance

S6.1. Suzuki reaction

Suzuki reaction with Pd-PDMS catalytic membrane

4-Bromotoluene (1.0×10^{-4} mol, 1.0 equiv), phenylboronic acid (1.1×10^{-4} mol, 1.1 equiv) and K_2CO_3 (1.5×10^{-4} mol, 1.5 equiv) were weighted in a 2 mL vial, and 0.8 mL of CD_3OD and 0.2 mL of D_2O was added. A pre-prepared catalytic membrane containing 1.7 mg of Pd-PDMS was placed in the vial. A gentle stream of argon was blown to the vial. The vial was tightly sealed and weighted. The reaction was carried out at 90 °C for 6 h under a constant stirring. The vial was weighted after completion of the reaction; no weight loss was detected. Afterwards 0.6 mL of the reaction mixture was placed an NMR tube and ^{1}H NMR spectrum was registered in order to calculate the yield of 4-methylbiphenyl. The yield of the target product was calculated by comparing integrated intensities of peaks corresponding to the methyl group of initial 4-iodoanisole and the target product. Yield of 4-methylbiphenyl was 80 %.

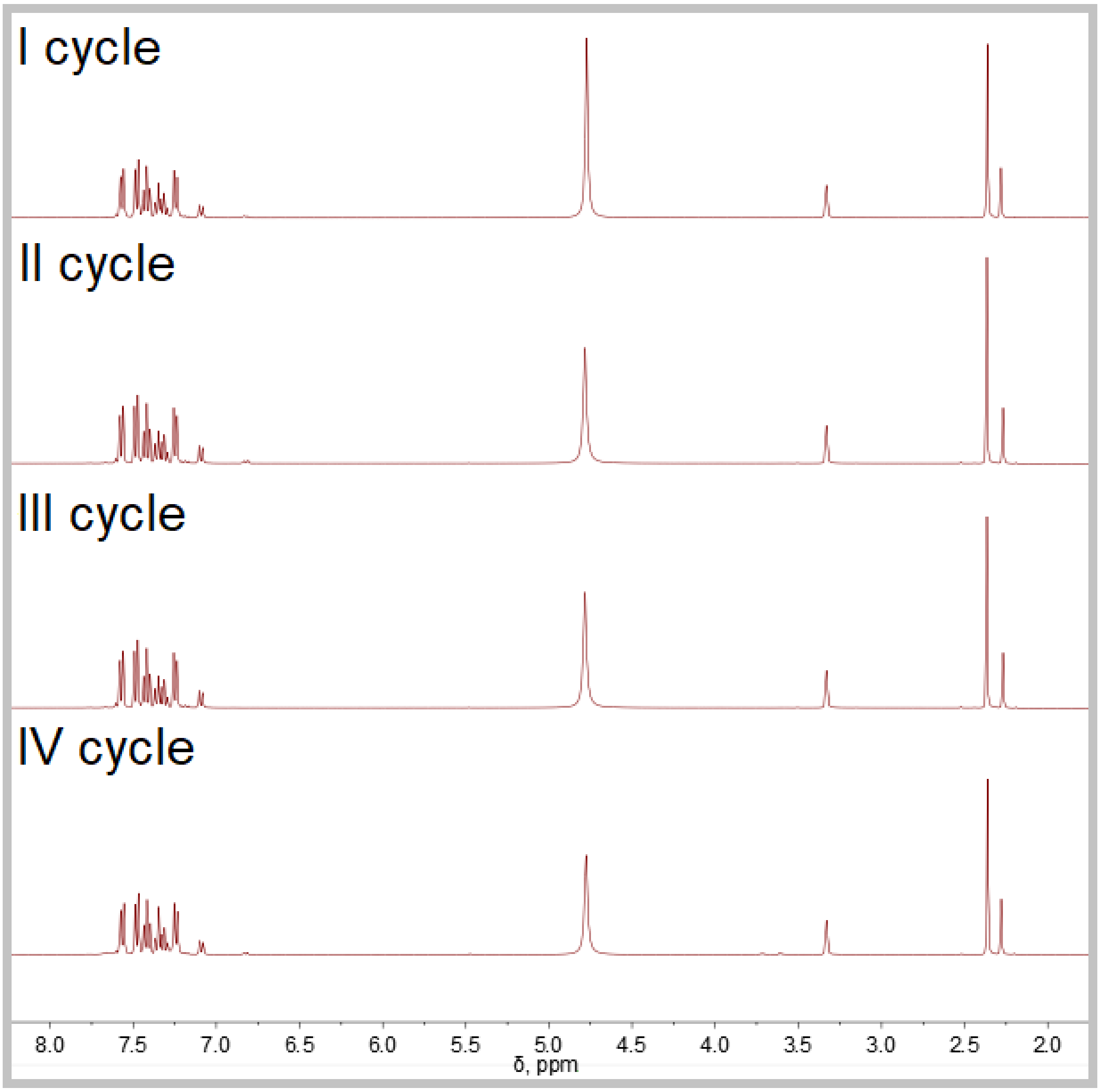

Figure S8. ^{1}H NMR of Suzuki coupling.

S6.2. Sonogashira reaction

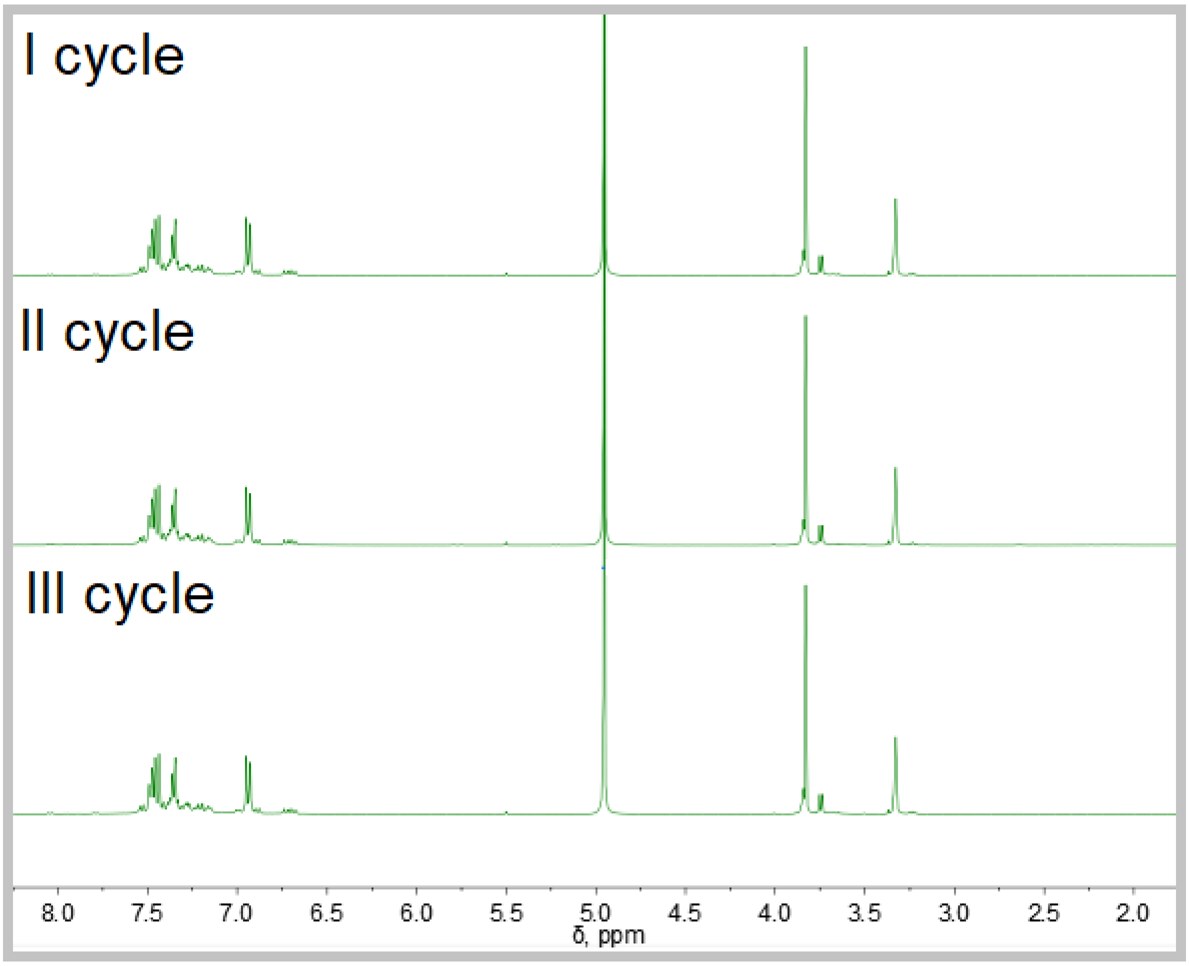

Figure S9. ^{1}H NMR of Sonogashira coupling.

S6.3. Heck reaction

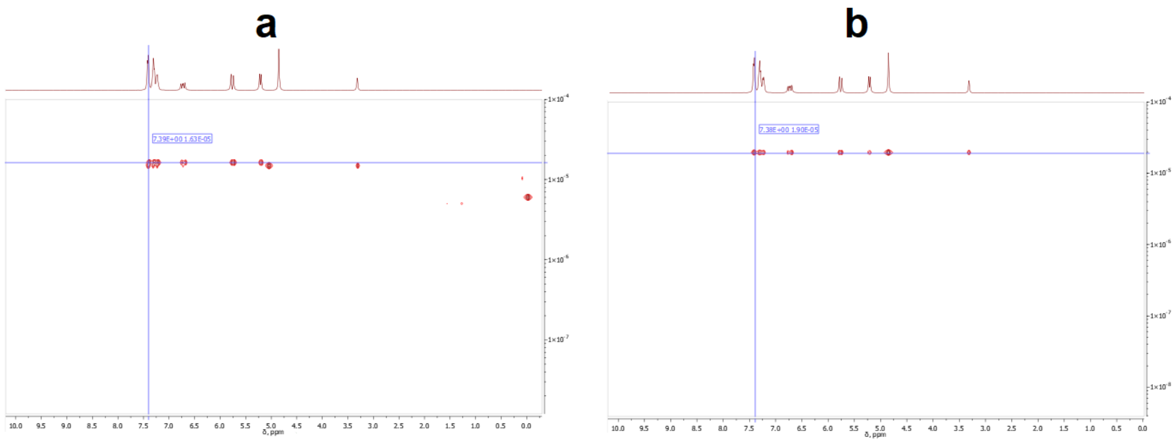

Figure S10. DOSY initial styrene (a) and after control test in Heck coupling conditions (b).

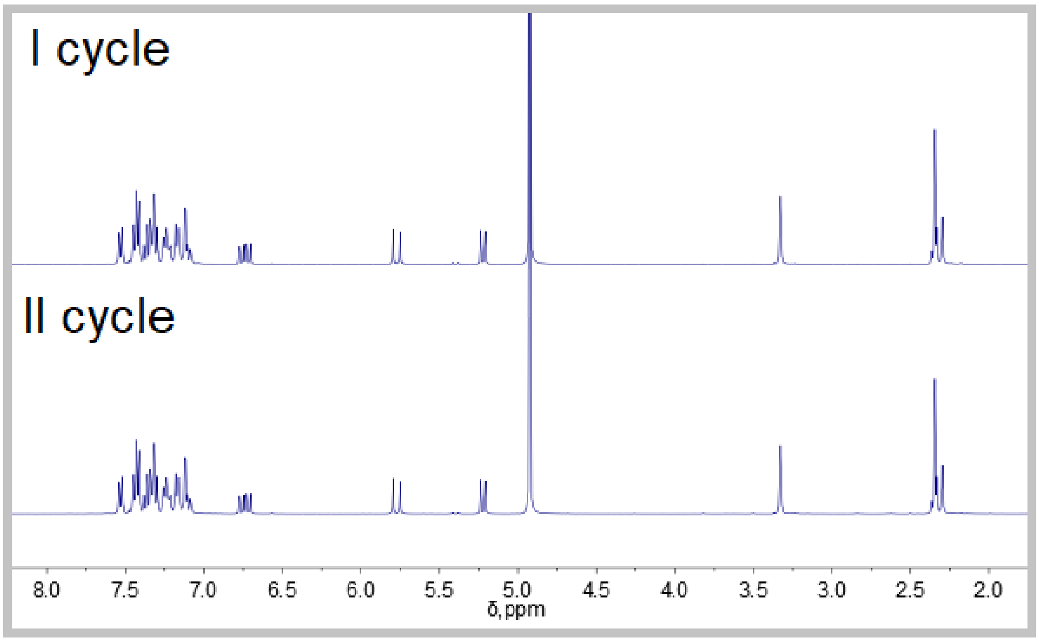

Figure S11. ^{1}H NMR of Heck coupling.

Table S1. The summary of the catalytic performance of Pd-PDMS catalytic membrane

Reaction	Yield* of the reaction product (mol %) in 1st run	Yield of the reaction product (mol %) in 2nd run	Yield of the reaction product (mol %) in 3rd run	Yield of the reaction product (mol %) in 4th run
Suzuki	80	80	80	70
Sonogashira	90	90	90	—
Heck	80	80	—	—

* On ^{1}H NMR data

S7. TON and TOF calculations

Turnover number (*TON*) for catalyst is the number of moles of substrate that a mole of catalyst can catalyze prior being inactive. TON was calculated using equation 1:

$$TON = \frac{n_{product}}{n_{catalyst}} = \frac{n_{\ substrate} \times X}{n_{catalyst}} \quad (1)$$

where *n* — the amount of mols; *X* — the product yield (molar fraction).

The turnover frequency (*TOF*) for catalyst is used to refer to the turnover per unit of time and calculated using equation 2:

$$TON = \frac{TON}{t} \quad (2)$$

where *t* — the reaction time, h.

Table S2. TON and TOF for Pd-PDMS

Reaction	TON	TOF, h^{-1}
Suzuki	800	133
Sonogashira	900	113
Heck	800	33

In the ref.[4] two palladium catalysts were obtained by cross-linking of triazolyl-containing polysiloxanes by $Pd(OAc)_2$. The obtained catalyst was used in Suzuki reactions. However, TON and TOF is not reported. In the ref.[5] palladium-containing catalyst was synthesized from $PdCl_2$(cod) with poly(3-N-imidazolopropyl)methylsiloxane-*co*-dimethylsiloxane). The catalyst demonstrated TOF up to 25,000 h^{-1} in the Suzuki couplings. However, the resulting catalyst was obtained in a form of beads and separation of the catalyst from the reaction mixture was difficult, even centrifugation was not efficient for the catalyst extraction from the reaction mixture.[6] At the same time Pd-PDMS is easy to apply in a form of a film on carbon support (other surface such as glass or zeolites is also applicable). The catalyst is easy to handle and used several times, which can be useful for a possible industrial application. In future the catalytic performance of the catalyst will be improved.

S8. DFT calculations

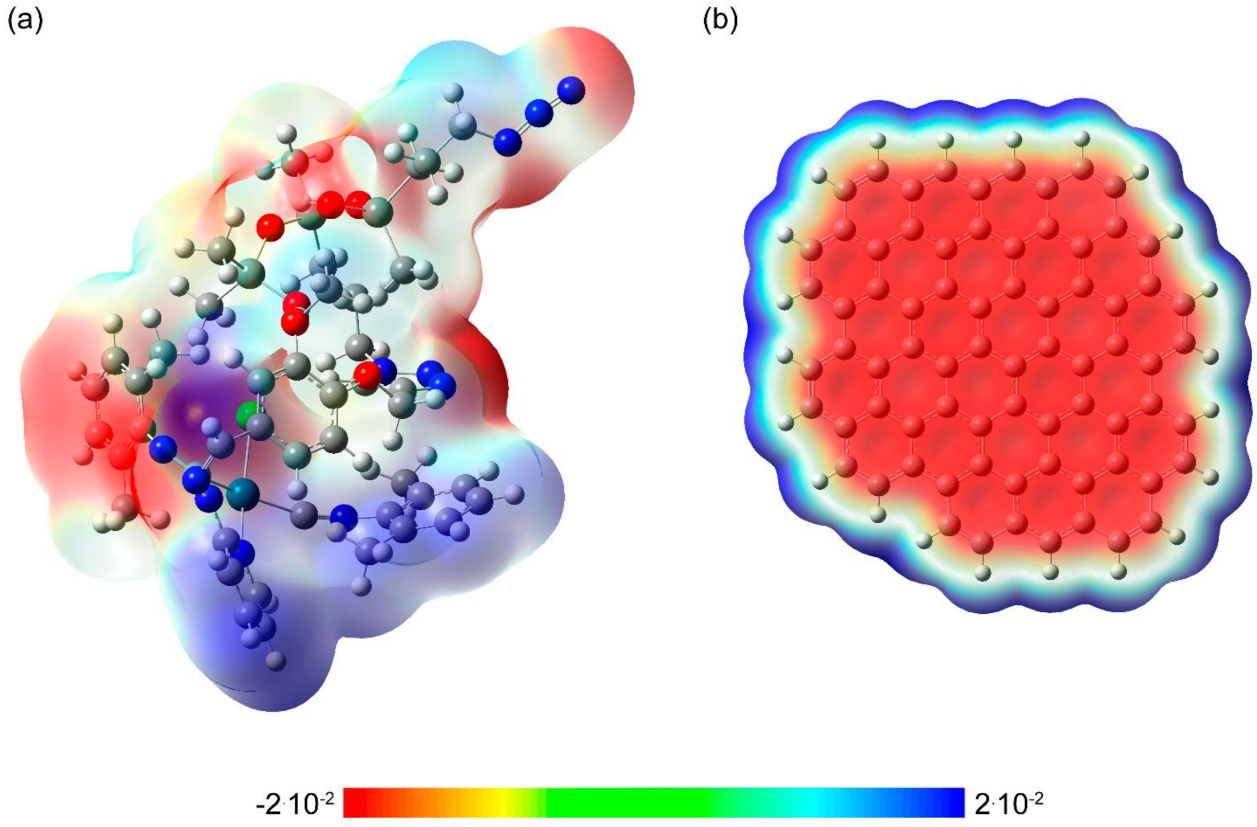

Figure S12. The geometry of Pd-PDMS (a) and PAH (b) and corresponding electrostatic potential maps.

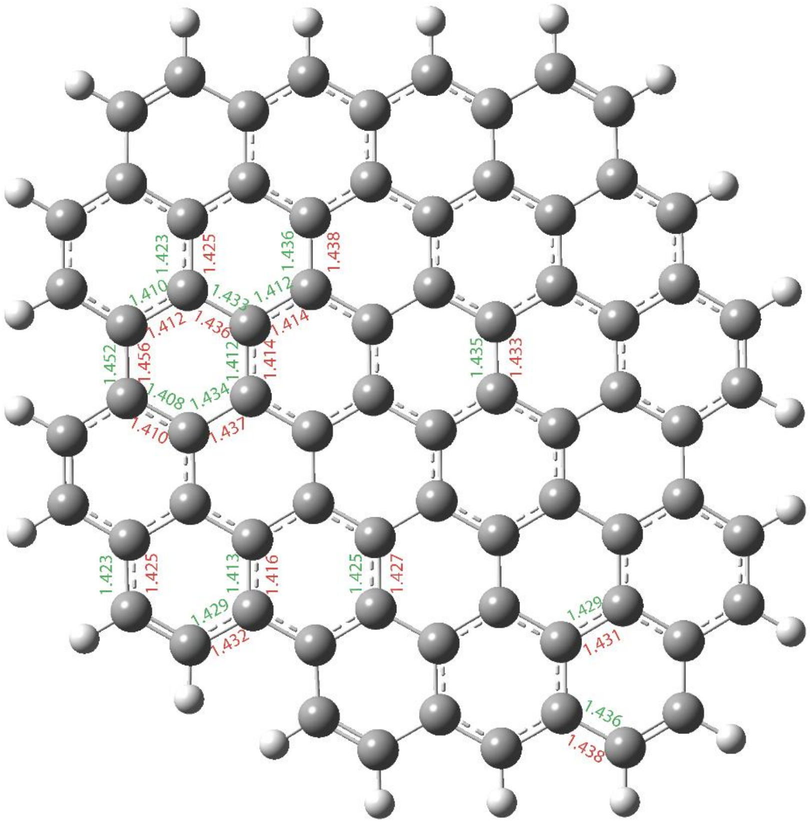

Figure S13. Bond lengths in PAH.

References:

(1) Cai, D.; Neyer, A.; Kuckuk, R.; Heise, H. M. Raman, Mid-Infrared, near-Infrared and Ultraviolet–Visible Spectroscopy of PDMS Silicone Rubber for Characterization of Polymer Optical Waveguide Materials. *J. Mol. Struct.* **2010**, *976* (1–3), 274–281. https://doi.org/10.1016/j.molstruc.2010.03.054.

(2) Talianov, P. M.; Rzhevskii, S. S.; Pankin, D. V.; Deriabin, K. V.; Islamova, R. M.; Manshina, A. A. Structural Features of Functional Polysiloxanes Radical and Ionic Photo-Curing for Laser Printing Applications. *J. Polym. Res.* **2021**, *28* (2), 37. https://doi.org/10.1007/s10965-021-02409-0.

(3) Durig, J. R.; Layton, R.; Sink, D. W.; Mitchell, B. R. Far Infrared Spectra of Palladium Compounds—I. The Influence of Ligands upon the Palladium Chloride Stretching Frequency. *Spectrochim. Acta* **1965**, *21* (8), 1367–1378. https://doi.org/10.1016/0371-1951(65)80046-7.

(4) Mieczyńska, E.; Borkowski, T.; Cypryk, M.; Pospiech, P.; Trzeciak, A. M. Palladium Supported on Triazolyl-Functionalized Polysiloxane as Recyclable Catalyst for Suzuki–Miyaura Cross-Coupling. *Appl. Catal. Gen.* **2014**, *470*, 24–30. https://doi.org/10.1016/j.apcata.2013.10.032.

(5) Borkowski, T.; Zawartka, W.; Pospiech, P.; Mizerska, U.; Trzeciak, A. M.; Cypryk, M.; Tylus, W. Reusable Functionalized Polysiloxane-Supported Palladium Catalyst for Suzuki–Miyaura Cross-Coupling. *J. Catal.* **2011**, *282* (2), 270–277. https://doi.org/10.1016/j.jcat.2011.06.023.

(6) Zawartka, W.; Pośpiech, P.; Cypryk, M.; Trzeciak, A. M. Palladium Supported on Aminopropyl-Functionalized Polymethylsiloxane Microspheres: Simple and Effective Catalyst for the Suzuki–Miyaura C–C Coupling. *J. Mol. Catal. Chem.* **2015**, *407*, 230–235. https://doi.org/10.1016/j.molcata.2015.07.002.
